# Anti-Vα24Jα18 TCR Antibody Tunes iNKT Cell Responses to Target and Kill CD1d-negative Tumors in an FcγRII (CD32)-dependent Manner

**DOI:** 10.1158/2767-9764.CRC-23-0203

**Published:** 2024-02-19

**Authors:** Mariko Takami, Takahiro Aoki, Katsuhiro Nishimura, Hidekazu Tanaka, Atsushi Onodera, Shinichiro Motohashi

**Affiliations:** 1 Department of Medical Immunology, Graduate School of Medicine, Chiba University, Chiba, Japan.; 2 Department of Pediatric Surgery, Graduate School of Medicine, Chiba University, Chiba, Japan.; 3 Department of Thoracic Surgery, Graduate School of Medical Sciences, Kumamoto University, Kumamoto, Japan.; 4 Institute for Advanced Academic Research, Chiba University, Chiba, Japan.; 5 Research Institute for Disaster Medicine, Chiba University, Chiba, Japan.

## Abstract

Invariant natural killer T (iNKT) cells play an essential role in antitumor immunity by exerting cytotoxicity and producing massive amounts of cytokines. iNKT cells express invariant T-cell receptors (TCR) to recognize their cognate glycolipid antigens such as α-galactosylceramide (α-GalCer) presented on CD1d. We recently reported that iNKT cells recognize CD1d-negative leukemia cell line K562 in a TCR-dependent manner. However, it remains controversial how iNKT cells use TCRs to recognize and exhibit cytotoxic activity toward CD1d-negative tumors cells without CD1d restriction. Here, we report that iNKT cells exerted cytotoxicity toward K562 cells via a carried over anti-Vα24 TCR mAb from positive selection by magnetic bead sorting. We found that addition of the anti-Vα24Jα18 TCR mAb (6B11 mAb) rendered iNKT cells cytotoxic to K562 cells in an FcγRII (CD32)-dependent manner. Moreover, iNKT cells treated with 6B11 mAb became cytotoxic to other CD32^+^ cell lines (U937 and Daudi). In addition, iNKT cells treated with 6B11 mAb suppressed K562 cell growth in a murine xenograft model *in vivo*. These data suggest that anti-iNKT TCR mAb treatment of iNKT cells can be applied as a therapeutic strategy to treat CD32^+^ cancers such as leukemia, lymphoma, and lung cancer.

**Significance:**

Our findings unveiled that iNKT cells recognize and kill CD1d-negative target tumors via the anti-iNKT TCR mAb bound to CD32 at the tumor site, thereby bridging iNKT cells and CD1d-negative tumors. These findings shed light on the therapeutic potential of anti-iNKT TCR mAbs in NKT cell–based immunotherapy to treat CD1d-negative CD32^+^ cancers.

## Introduction

Invariant natural killer T (iNKT) cells are an unconventional T-cell subset that express an invariant T-cell receptor (TCR; Vα24 and Vβ11 in humans, and Vα14, Vβ8.2, Vβ2, and Vβ7 in mice) and recognize glycolipid antigen α-galactosylceramide (α-GalCer) presented on CD1d, followed by activation events (1, 2). Activated iNKT cells produce massive amounts of cytokines, such as IFNγ and TNFα, and exert cytotoxicity by producing granzymes and perforin. iNKT cells play an essential role in antitumor immunity by directly targeting tumors or indirectly activating NK cells or cytotoxic CD8^+^ T cells. Nevertheless, very few iNKT cells (<0.1%) are present in the periphery in humans (3, 4).

iNKT cell–based immunotherapy has been developed to treat various cancers including lung cancer, head and neck cancer, neuroblastoma, and melanoma (5–9). We previously reported that α-GalCer–pulsed antigen-presenting cell (APC) administration, which activates iNKT cells, prolongs overall survival of patients with non–small cell lung cancer (10). α-GalCer–pulsed APCs induce activation of iNKT cells and increase IFNγ-producing cells. However, it is unknown whether iNKT cells directly recognize and kill target tumors upon α-GalCer–pulsed APC administration.

We and others have shown that iNKT cells recognize and kill CD1d-negative target cells including K562 cells (11). Natural killer (NK) cell–activating receptors, such as NKG2D, DNAM1, and 2B4, and costimulatory molecules LFA-1 and LFA-2 are involved in this process (11, 12). However, some studies have suggested that iNKT cells recognize target cells only when glycolipid ligand is present along with CD1d expression. Therefore, iNKT cells cannot exert cytotoxic activity toward CD1d-negative K562 cells (5). We recently reported that knocking out the TCR α- or β-chain in iNKT cells by the CRISPR/Cas9 system abrogates the cytotoxic activity of iNKT cells toward CD1d-negative K562 cells, indicating that invariant TCRs are required to recognize CD1d-negative K562 cells (11). Because invariant Vα24Vβ11 TCRs recognize their cognate glycolipid antigen with CD1d restriction, it remains unclear how iNKT cells use TCRs to recognize K562 cells without CD1d expression. In this study, we investigated the mechanisms by which iNKT cells exert cytotoxicity toward CD1d-negative K562 cells in a TCR-dependent manner. We found that invariant TCRs were required for cytotoxic activity toward K562 cells. However, invariant TCRs were not directly recognizing target cells. We revealed that a carried over anti-Vα24 mAb from positive selection by magnetic bead sorting were attached to TCRs of iNKT cells. Moreover, the FC portion of the mAb bound to FCγ receptor II (CD32) expressed on K562 cells induced crosslinking of TCRs of iNKT cells, thereby inducing cytotoxic activity toward K562 cells. Anti-Vα24-Jα18 mAb (6B11 mAb) treatment of iNKT cells induced cytotoxic activity toward CD1d-negative K562 cells *in vitro* and *in vivo* in a CD32-dependent manner.

## Materials and Methods

### Cell Lines

K562 (CCL-243, RRID: CVCL_0004), U937 (CRL-1593.2, RRID: CVCL_0007), Daudi (CCL-213, RRID: CVCL_0008), and Jurkat (TIB-152, RRID: CVCL_0065) cells were purchased from the ATCC (https://www.atcc.org). A549 (RRID: CVCL_0023) and PC13 cells (RRID: CVCL_B259) were a gift from Dr. Kazuo Suzuki. Cell lines were cultured in RPMI1640 medium (FUJIFILM) supplemented with 10% FCS, 100 U/mL penicillin G, 100 µg/mL streptomycin (Thermo Fisher Scientific), and 10 mmol/L Hepes (Thermo Fisher Scientific). Cells were routinely tested for *Mycoplasma* contamination using Mycostrip (Invivogen). K562 and A549 cells were authenticated through short tandem repeat profiling on February 1, 2023 (BEX Co., Ltd.). Reauthentication for other cell lines was not performed. Cell lines were passaged less than 12 times after thawing.

### Cell Preparation and Culture

Peripheral blood mononuclear cells (PBMC) were isolated from whole blood of healthy donors by density gradient centrifugation using Ficoll Paque Plus (Cytiva). iNKT cells were expanded from PBMCs in complete RPMI1640 medium (RPMI1640 medium with 10% FCS, 100 U/mL penicillin G, 100 µg/mL streptomycin, 200 µmol/L L-alanyl-L-glutamine; FUJIFILM, 50 µmol/L 2-mercaptoethanol; Thermo Fisher Scientific, 10 mmol/L Hepes, MEM non-essential amino acids; Thermo Fisher Scientific, MEM Essential Amino Acids Solution; FUJIFILM, 1 mmol/L sodium pyruvate; FUJIFILM) containing IL2 (100 U/mL, Shionogi) and α-GalCer (200 ng/mL, REGiMMUNE) for 9–10 days. Expanded iNKT cells were labeled with an anti-Vα24 FITC antibody (Beckman Coulter) and purified using FITC MicroBeads (Miltenyi Biotec). Sorted iNKT cells were cultured in complete RPMI1640 medium with IL2 (100 U/mL) until analysis. For Ab treatment of iNKT cells, sorted iNKT cells were cultured in complete RPMI1640 medium with IL2 for 20 hours, washed twice, and then cultured in fresh complete RPMI1640 medium with IL2 for 4–5 days. iNKT cells were treated with 6B11, anti-CD3ε, anti-CD2, and anti-CD28 mAbs (BioLegend) for 20 hours and then washed with fresh medium prior to coculture with cell lines. For blocking experiments, 6B11mAb-bound iNKT cells were pretreated with an anti-CD32 mAb (10 µg/mL, Thermo Fisher Scientific), anti-CD64 mAb (10 µg/mL, BioLegend), anti-NKG2D mAb (10 µg/mL, BioLegend), anti-DNAM-1 mAb (10 µg/mL, R&D Systems), anti-2B4 mAb (10 µg/mL, Thermo Fisher Scientific), anti-CD11a (LFA-1) mAb (10 µg/mL, BioLegend), anti-CD2 (LFA-2) mAb (10 µg/mL, BioLegend), or isotype control mIgG1 (10 µg/mL, BioLegend) for 30 minutes. Cells were then cocultured with K562 cells for a CD107a assay. For iNKT cell stimulation, iNKT cells were stimulated in flat-bottom 96-well plates coated with 6B11 or anti-CD3ε mAbs at the indicated concentrations for a CD107 assay. For cross-linking experiments, 6B11 mAb-bound iNKT cells were incubated with goat anti-mouse IgG (10 µg/mL Jackson ImmunoResearch) for the CD107 assay. Normal cells used as targets of iNKT cells were isolated from whole blood or autologous PBMCs of healthy donors. B cells were labeled with an anti-CD19 FITC Ab and purified from PBMCs using FITC MicroBeads (Miltenyi Biotec). Monocytes were purified from PBMCs using CD14 MicroBeads (Miltenyi Biotec). Neutrophils were isolated from healthy donor blood using a MojoSort Whole blood Neutrophil Isolation kit (BioLegend). Cell sorting procedures were performed by following the manufacturers’ protocols.

### Collection of Culture Supernatant and Mouse Ig Depletion

Sorted iNKT cells were seeded in 6-well plates (7.5 × 10^5^ cells/mL) in complete RPMI1640 medium with IL2 (100 U/mL) and cultured for 20–24 hours. Culture supernatants were collected and stored at −80°C until analysis. For mouse Ig depletion, Dynabeads Sheep-Anti-Mouse IgG (Thermo Fisher Scientific) were used following the manufacturer's protocol. Briefly, prewashed Dynabeads Sheep-Anti Mouse IgG were incubated with culture supernatant (4 × 10^7^ beads/mL) for 30 minutes at room temperature with gentle rocking and then removed using a magnetic stand.

### Flow Cytometry

Cells were resuspended in FACS buffer (1 × PBS with 1% FCS and 0.1% sodium azide) and stained with fluorochrome-conjugated Abs for 30 minutes at 4°C. Dead cells were excluded using a Zombie Aqua Fixable Viability Kit (BioLegend). The following Abs were used for staining. Vα24-FITC and Vβ11-PE mAbs were purchased from Beckman Coulter. A CD1d-APC mAb was purchased from eBioScience. CD16-Pacific Blue, CD32-PE, CD64-APC, and CD3-Pacific Blue mAbs were purchased from BioLegend. Data were collected on a BD LSRFortessa (BD Biosciences) and analyzed using FlowJo software (BD Biosciences).

### Measurement of Cytotoxicity

For the CD107a assay, iNKT cells were cocultured with K562 cells or other cell lines at an effector to target (E/T) ratio of 2:1 in the presence of monensin and a PE-labeled anti-CD107a Ab (BioLegend) for 2 hours. Cells were then stained with anti-Vα24-FITC and anti-CD3-Pacific Blue Abs to identify iNKT cells followed by flow cytometric analysis to assess surface expression of CD107a on iNKT cells.

For the lactate dehydrogenase (LDH) cytotoxicity assay, iNKT cells were cocultured with K562 cells at E/T ratios of 10:1, 5:1, and 2:1 for 4 hours. LDH release was detected using a Cytotoxicity Detection Kit plus (LDH; Roche) as described in the manufacturer's protocol. Percentage cytotoxicity was calculated by the formula % cytotoxicity = 100 × [(experimental value − spontaneous cell death)/(maximum cell death − spontaneous cell death)].

### Measurement of Cytokine Release

iNKT cells were cocultured with the indicated cell lines at an E/T ratio of 2:1 for 6 hours. Culture supernatants were collected and production of IFNγ and TNFα was assessed by a BD Cytometric Bead Array (BD Biosciences). Data were collected using FACSVerse (BD Biosciences) and analyzed by FCAP Array Software v3.0 (BD Biosciences).

### Lentiviral Transduction

Lentiviral vector pLV[Exp]-Bsd-EF1A>hFCGR2A was constructed and packaged by VectorBuilder to overexpress CD32 under the control of the EF-1α promoter. A549 cells were transduced with 0.1 or 1 multiplicity of infection of the lentivirus by spinoculation at 400 × *g* for 2 hours in the presence of polybrene (5 µg/mL). After 7 days of transduction, A549 cells expressing CD32 at various levels were sorted into single cells by a BD FACSAria II Cell Sorter (BD Biosciences).

### IHC

Lung tumor tissue arrays containing sections from 64 donors were obtained from Biochain. Sections were deparaffinized, and endogenous peroxidase activity was quenched by incubation in 3% H_2_O_2_. Sections were then incubated with an anti-CD32-A/B/C Ab (Santa Cruz Biotechnology) at 4°C overnight, followed by incubation with an horseradish peroxidase–conjugated anti-mouse IgG Ab (Nichirei Biosciences Inc.) at room temperature for 30 minutes. Sections were further incubated with 3,3ʹ-diaminobenzidine for development and counterstained with Mayer's hematoxylin. Stained sections were evaluated by microscopy and scored by the proportion of CD32^+^ tumor cells: score 1, <10% positive cells; score 2, 10%–50% positive cells; score 3, >50% positive cells. All IHC procedures were outsourced and performed by Kyodo Byori Inc.

### *In Vivo* Mouse Experiments

hIL7 × hIL15 KI NOD/SCID/IL2rgKO (NSG) mice were kindly provided by Drs. Koseki and Ishikawa, RIKEN (13). Mice (male or female) were subcutaneously injected with K562 cells (2 × 10^6^ cells in 50 µL of PBS) mixed with 50 µL Matrigel Matrix (Corning) on day 0. On day 3, the tumor size was measured, and mice injected with K562 cells were divided into each group by the tumor size. Treatments were performed on days 3, 5, 7, and 9 as indicated: group 1, mice were intratumorally injected with 50 µL PBS; group 2, mice were intratumorally injected with 2 × 10^6^ iNKT cells pretreated with mIgG1 (50 ng/mL) in 50 µL PBS; group 3, mice were intratumorally injected with 2 × 10^6^ iNKT cells pretreated with 6B11 mAb (50 ng/mL) in 50 µL PBS; group 4, mice were intravenously injected with 2 × 10^6^ iNKT cells pretreated with 6B11 mAb (50 ng/mL, Thermo Fisher Scientific) in 200 µL PBS; group 5, mice were intratumorally injected with 2 × 10^6^ iNKT cells in 200 µL PBS and intraperitoneally injected with 50 µg 6B11 mAb (in 100 µL PBS). The tumor size was monitored using a caliper every other day after day 5. The tumor volume was calculated by the formula *V* = (*W*^2^ × *L*)/2.

### Gene Expression Profiling Interactive Analysis

The Gene Expression Profiling Interactive Analysis (GEPIA) database (http://gepia2.cancer-pku.cn/#index), which is an RNA-sequencing data platform that includes multiple tumor tissues from The Cancer Genome Atlas (TCGA) database, was used to analyze expression of the *FCGR2A* gene, which encodes CD32 across tumors. We chose 12 tumors for the analysis.

### Statistical Analysis

Statistics were calculated using GraphPad Prism 7.0 (GraphPad Software). For *in vitro* experiments, the unpaired two-tailed Student *t* test was used to evaluate two groups and one-way ANOVA with Tukey multiple comparisons test was used to evaluate more than two groups. For *in vivo* tumor growth experiments, two-way ANOVA with Tukey multiple comparisons test was used to evaluate three groups.

### Data Availability

The data generated in this study are available in the article.

### Study Approval

Whole blood from healthy donors and bone marrow from patients with acute myeloid leukemia (LAML) were collected with written informed consent in accordance with protocols approved by the Institutional Ethics Committee (#3455 and #HK202308-10). The study was conducted in accordance with the Declaration of Helsinki. Animal experiments were approved by the Institutional Animal Care and Use Committee of Chiba University (Chiba, Japan).

## Results

### Soluble Factors in iNKT Cell Cultures Mediate Degranulation of iNKT Cells Toward K562 Cells

**FIGURE 1:**
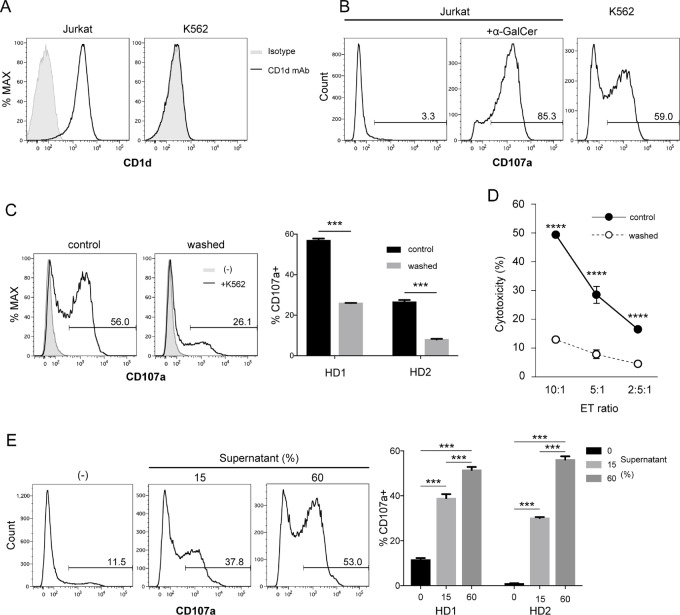
Soluble factors in iNKT cell cultures mediate degranulation of iNKT cells toward K562 cells. **A,** Jurkat and K562 cells were stained with an anti-CD1d Ab and analyzed by flow cytometry. **B–E,** PBMCs isolated from healthy donors were cultured in the presence of α-GalCer and IL2 for 9–10 days, followed by sorting using Vα24-FITC Ab/FITC MicroBeads. iNKT cells were then treated with IL2 until analysis. **B,** iNKT cells maintained for 3 days after sorting were cocultured with K562, Jurkat, or α-GalCer–pulsed Jurkat cells at an E/T ratio of 2:1 and then a CD107a assay was performed. **C** and **D,** iNKT cells maintained overnight after sorting were washed and cultured in fresh medium or maintained in the same medium (control) for 2 days. **C,** iNKT cells were cocultured with K562 cells at an E/T ratio of 2:1 and then a CD107a assay was performed. **D,** iNKT cells were cocultured with K562 cells at 10:1, 5:1, and 2.5:1 E/T ratios. An LDH release assay was performed and the percentage cytotoxicity was calculated. **E,** Sorted iNKT cells were maintained overnight and then the culture supernatant was collected. iNKT cells maintained overnight after sorting were washed and then maintained for 4 days. The culture supernatant was added to an iNKT cell culture overnight prior to coculture with K562 cells for the CD107a assay. Data are representative of two independent experiments. Data represent the mean ± SEM. ***, *P* < 0.001; ****, *P* < 0.0001. HD, healthy donor.

iNKT cells recognize α-GalCer presented on CD1d via TCRs. However, it remains controversial whether CD1d is required to recognize tumors. We assessed cell surface expression of CD1d on Jurkat and K562 cells. Jurkat cells expressed a high level of CD1d, whereas no expression of CD1d was detected on K562 cells as reported previously (Fig. 1A; ref. 11). To assess cytotoxic activity of iNKT cells toward CD1d^+^ Jurkat and CD1d-negative K562 cells, we assessed surface expression of CD107a (Lamp1), which is an indicator of degranulation, by flow cytometry. When MACS-sorted iNKT cells rested for 3 days were cocultured with Jurkat cells, CD107a^+^ iNKT cells were barely detectable, while CD107a^+^ iNKT cells were drastically increased with α-GalCer–pulsed Jurkat cells (Fig. 1B). These results indicated that iNKT cells exhibited cytotoxicity in a CD1d-dependent manner only after exogenously adding the ligand α-GalCer. However, iNKT cells also underwent degranulation toward CD1d-negative K562 cells without α-GalCer pulsing, indicating that iNKT cells exerted cytotoxicity regardless of the presence or absence of CD1d expression on tumors. Because we empirically found that the cytotoxic activity of iNKT cells toward K562 cells was the highest 2 days after MACS sorting and gradually decreased daily (Supplementary Fig. S1), we determined whether soluble factors produced by iNKT cells after MACS sorting mediated cytotoxic activity toward K562 cells. To this end, iNKT cells were washed at 1 day after MACS to remove soluble factors from the culture supernatant and then maintained in fresh medium for 2 days. CD107a^+^ iNKT cells after coculture with K562 cells were drastically decreased when iNKT cells were washed compared with control iNKT cells without washing (Fig. 1C). To confirm K562 cell killing by iNKT cells, we performed an LDH-based cytotoxicity assay. Washed iNKT cells exhibited less cytotoxicity compared with control iNKT cells without washing (Fig. 1D). We next determined whether addition of culture supernatant converted iNKT cells, which did not degranulate toward K562 cells to exert cytotoxic activity. iNKT cells were washed at 1 day after MACS and maintained in fresh medium for 3 days. iNKT cells treated with culture supernatant for 20 hours were cocultured with K562 cells to assess degranulation activity. Whereas control iNKT cells without culture supernatant treatment were 11.5% CD107^+^ after coculture with K562 cells, CD107^+^ iNKT cells had increased to 37.8% under 15% culture supernatant treatment and 53% with 60% culture supernatant treatment (Fig. 1E).

### 6B11 mAb Treatment Induces Degranulation of iNKT Cells Toward K562 Cells

**FIGURE 2:**
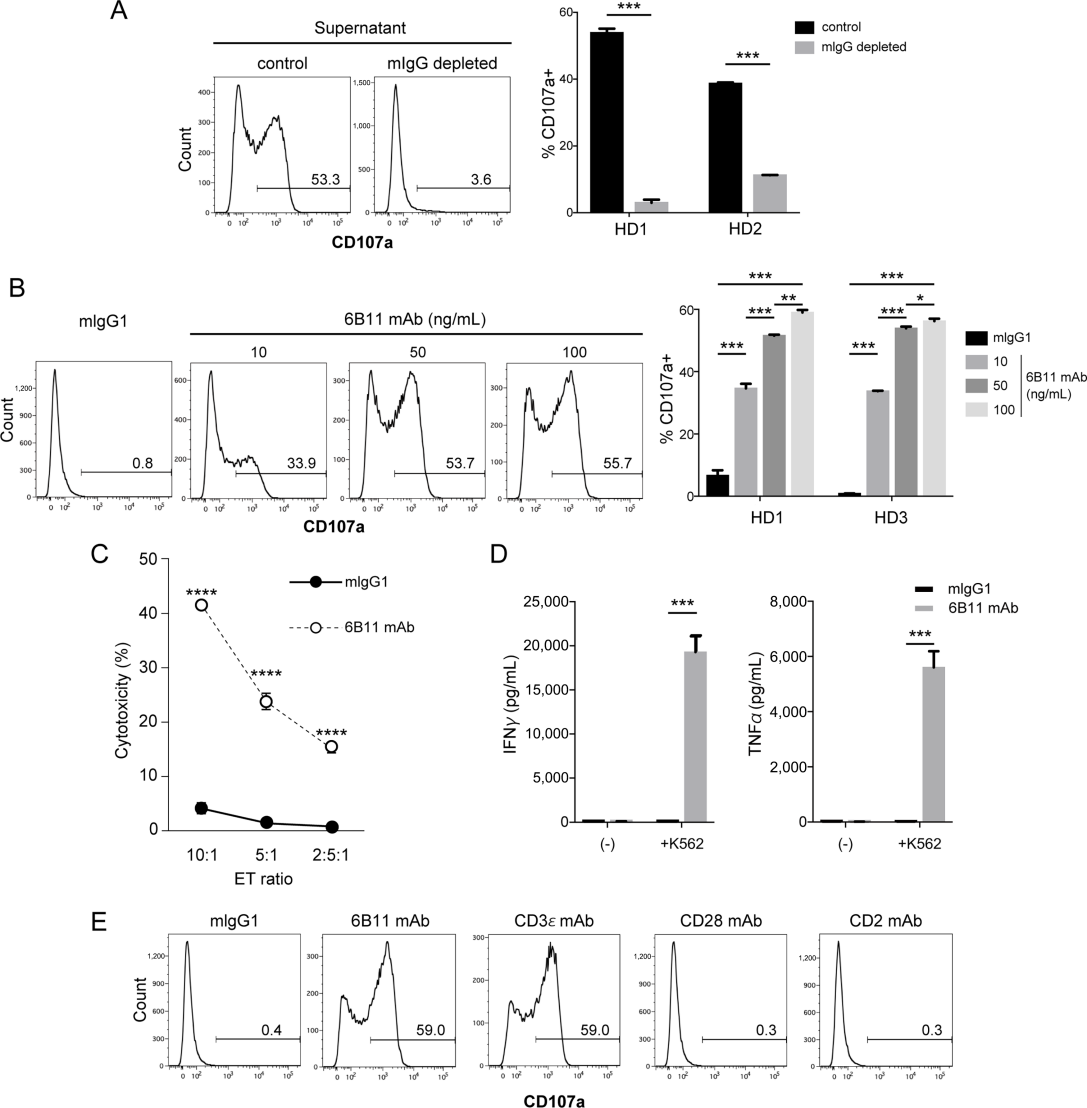
6B11 mAb induces degranulation of iNKT cells toward K562 cells. PBMCs isolated from healthy donors were cultured in the presence of α-GalCer and IL2 for 9–10 days, followed by sorting using Vα24-FITC Ab/FITC MicroBeads. Sorted iNKT cells were maintained in the presence of IL2 overnight and then washed. iNKT cells were then maintained in the presence of IL2 until analysis. **A,** Sorted iNKT cells were maintained overnight and then the culture supernatant was collected. The culture supernatant depleted with anti-mouse Ig beads or the control was added to iNKT cell cultures overnight prior to coculture with K562 cells for the CD107a assay. **B,** Sorted iNKT cells treated with the 6B11 mAb (10, 50, and 100 ng/ mL) or isotype mAb (mIgG1, 50 ng/mL) were cocultured with K562 cells for CD107a assays. **C,** Sorted iNKT cells treated with the 6B11 mAb (50 ng/mL) or isotype mAb (50 ng/mL) were cocultured with K562 cells at E/T ratios of 10:1, 5:1, and 2.5:1. An LDH release assay was then performed. **D,** Sorted iNKT cells treated with the 6B11 mAb (50 ng/ mL) or isotype (mIgG1, 50 ng/mL) were cocultured with K562 cells. Culture supernatants were collected for Cytometric Bead Array to assess IFNγ and TNFα production. **E,** iNKT cells were treated with the 6B11 mAb and anti-CD3e (UCHT1), anti-CD28, anti-CD2, or isotype control Abs overnight. iNKT cells were then washed and cocultured with K562 cells. Data are representative of two independent experiments. Data represent the mean ± SEM. *, *P* < 0.05; **, *P* < 0.01; ***, *P* < 0.001; ****, *P* < 0.0001. HD, healthy donor.

Although iNKT cells were washed twice after MACS, the Vα24-FITC mAb was considered to remain attached to iNKT cells after positive selection. Because iNKT cells require TCRs to recognize K562 cells via unknown mechanisms as we reported previously, we hypothesized that the culture supernatant contained anti-Vα24 mAb shed from iNKT cells sorted by anti-Vα24 mAb-FITC/FITC beads and this anti-Vα24 mAb was the mediator of cytotoxic activity toward CD1d-negative K562 cells. To determine whether the culture supernatant contained carried over the anti-Vα24 FITC mAb from MACS, which mediated degranulation of iNKT cells toward K562 cells, we depleted the anti-Vα24-FITC mAb using anti-mouse IgG microbeads. iNKT cells treated with mouse IgG-depleted culture supernatant lost their ability to degranulate toward K562 cells, whereas iNKT cells treated with control culture supernatant exhibited cytotoxic activity, indicating that iNKT cells exhibited tumoricidal activity in an anti-Vα24 FITC mAb-dependent manner (Fig. 2A). Next, we determined whether anti-Vα24 mAb treatment was sufficient to degranulate iNKT cells toward K562 cells. To this end, we pretreated iNKT cells with 10, 50, or 100 ng/mL 6B11 mAb, which recognized the Vα24Jα18 CDR3 loop clonotypic TCR, and assessed the degranulation activity of iNKT cells toward K562 cells (14). Surface expression of CD107a on iNKT cells was increased in a 6B11 mAb dose-dependent manner (Fig. 2B). We next assessed the cytotoxic activity of iNKT cells treated with 6B11 mAb by an LDH release assay to confirm whether target K562 cells were indeed killed by iNKT cells treated with the 6B11 mAb. iNKT cells treated with the 6B11 mAb exhibited strong cytotoxicity toward K562 cells, whereas isotype mAb-treated iNKT cells exhibited a basal level of cytotoxicity (Fig. 2C). iNKT cells treated with the 6B11 mAb also produced large amounts of antitumor cytokines IFNγ and TNFα toward K562 cells (Fig. 2D). To address whether treatment with antibodies targeting other molecules in the central-supramolecular activation complex also made iNKT cells cytotoxic to CD1d-negative K562 cells, we treated iNKT cells with anti-CD3ε (UCHT1), anti-CD28, or anti-CD2 mAbs and assessed degranulation toward K562 cells. iNKT cells treated with the anti-CD3ε mAb showed high CD107a expression, but degranulation of iNKT cells was not induced by anti-CD28 or anti-CD2 mAb treatment (Fig. 2E). These data suggested that iNKT cells can target CD1d-negative K562 cells in a mAb-targeting TCR complex–dependent manner.

### 6B11 mAb Treatment Induces Degranulation of iNKT Cells Toward Some Cell Lines

**FIGURE 3:**
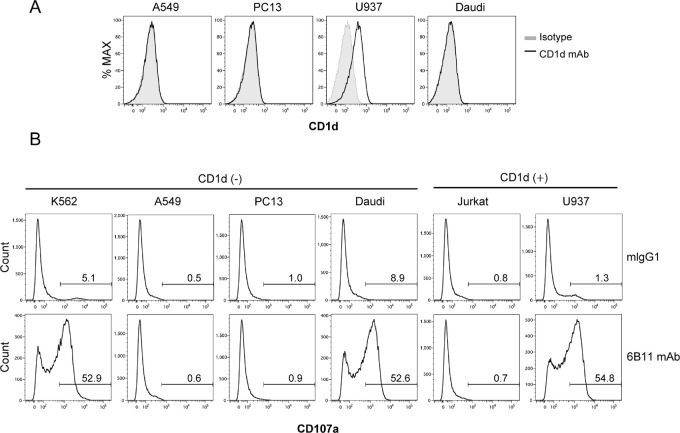
6B11 mAb treatment induces degranulation of iNKT cells toward some cell lines. **A,** The indicated cell lines were stained with an anti-CD1d mAb and analyzed by flow cytometry. **B,** PBMCs isolated from healthy donors were cultured in the presence of α-GalCer and IL2 for 9–10 days, followed by sorting using Vα24-FITC Ab/FITC MicroBeads. iNKT cells sorted by MACS were maintained in the presence of IL2 overnight, and then iNKT cells were washed twice and cultured in fresh medium with IL2 for 4–5 days. iNKT cells treated with the 6B11 mAb (50 ng/mL) or isotype (mIgG1) were cocultured with the indicated cell lines at an E/T ratio of 2:1. A CD107a assay was then performed. Data are representative of two independent experiments.

Thus far, we addressed cytotoxic activity of iNKT cells treated with a 6B11 mAb toward CD1d-negative K562 cells. We next examined whether 6B11 mAb treatment rendered iNKT cells cytotoxic to other cancer cell lines regardless of CD1d expression. A549, PC13, and Daudi cells did not express CD1d, whereas U937 cells did express CD1d (Fig. 3A). iNKT cells treated with the 6B11 mAb degranulated toward CD1d-negative Daudi and CD1d^+^ U937 cells, but not toward CD1d-negative A549, CD1d-negative PC13, or CD1d^+^ Jurkat cells, indicating that CD1d expression on tumor cells was not required for this antitumor activity of iNKT cells induced by 6B11 mAb treatment (Fig. 3B). These data suggested that iNKT cells treated with the 6B11 mAb were cytotoxic to some cell lines, but not all.

### 6B11 mAb Treatment Induces Degranulation of iNKT Cells Toward K562 Cells in a CD32-dependent Manner

**FIGURE 4:**
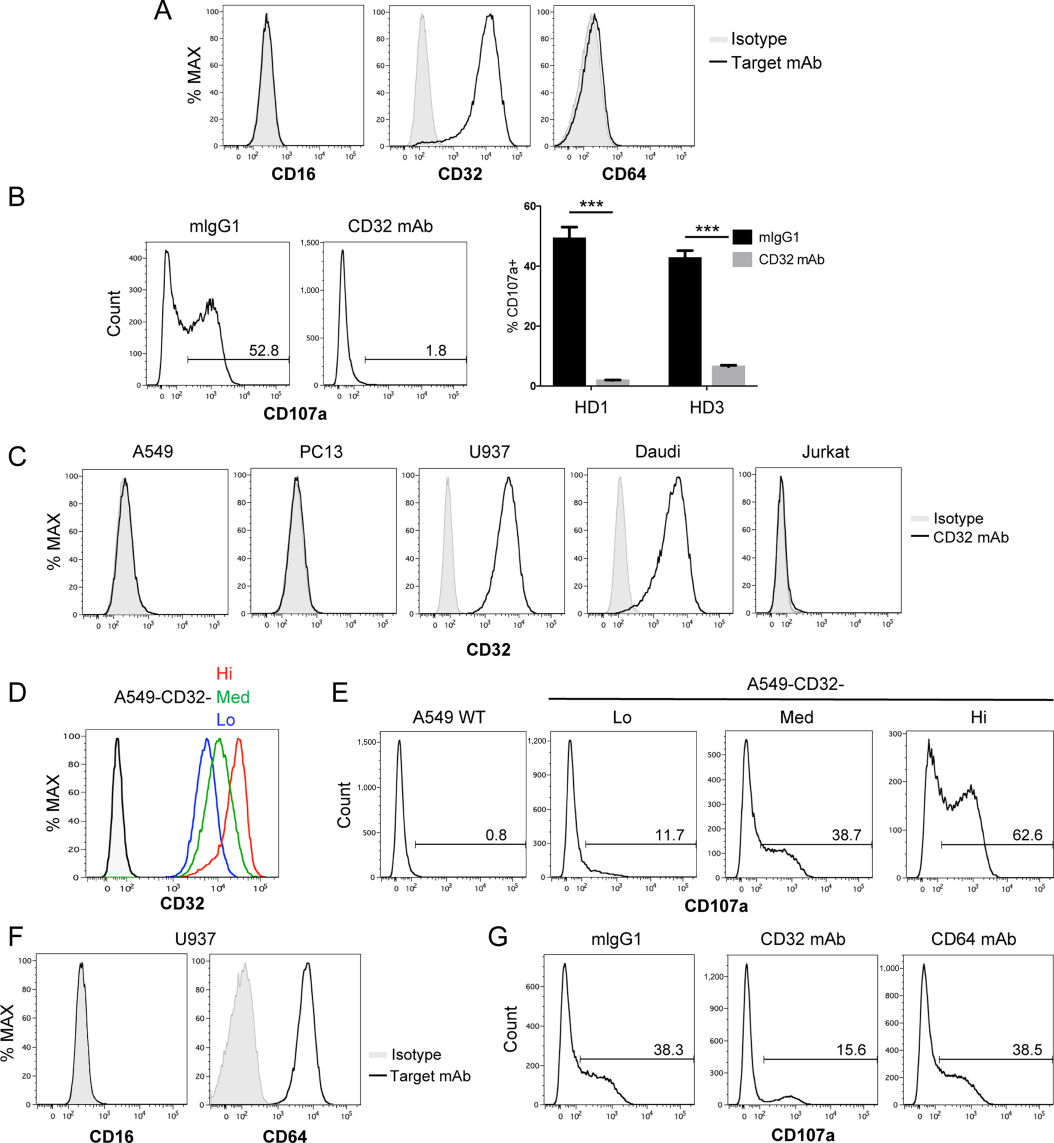
6B11 mAb treatment induces degranulation of iNKT cells toward K562 cells in a CD32-dependent manner. **A,** K562 cells were stained with anti-FC receptor (CD16, CD32, and CD64) mAbs and assessed by flow cytometry. **B, E, G,** iNKT cells sorted by using Vα24-FITC Ab/FITC MicroBeads were maintained in the presence of IL2 overnight, and then iNKT cells were washed twice and cultured in fresh medium with IL2 for 4–5 days. **B,** iNKT cells treated with the 6B11 mAb (50 ng/mL) overnight were washed and cocultured with K562 cells at an E/T ratio of 2:1 in the presence of an anti-CD32 mAb or isotype (mIgG1, 10 µg/mL). An CD107a assay was then performed. **C,** The indicated cell lines were stained with an anti-CD32 mAb and assessed by flow cytometry. **D,** CD32 was constitutively overexpressed via lentiviral transduction in A549 cells. A549 cells, which express CD32 at various levels, were stained with an anti-CD32 mAb and then assessed by flow cytometry. **E,** iNKT cells treated with the 6B11 mAb (50 ng/mL) overnight were washed and cocultured with A549 cells overexpressing CD32 at an E/T ratio of 2:1. A CD107a assay was then performed. **F,** U937 cells were stained with anti-CD16 and anti-CD64 mAbs and then assessed by flow cytometry. **G,** iNKT cells treated with the 6B11 mAb (50 ng/mL) overnight were washed and cocultured with U937 cells at an E/T ratio of 2:1 in the presence of anti-CD32, anti-CD64, or isotype control Abs. A CD107a assay was then performed. Data are representative of two independent experiments. Data represent the mean ± SEM. ***, *P* < 0.001. HD, healthy donor.

To decipher the mechanism by which 6B11 mAb treatment induced cytotoxicity of iNKT cells toward CD1d-negative K562 cells, we hypothesized that FC receptors (CD16, CD32, and CD64) expressed in K562 cells might bridge 6B11 mAb-treated iNKT cells and K562 cells, thereby cross-linking TCRs on iNKT cells and triggering cytotoxic activity toward K562 cells. To test this hypothesis, we examined expression of CD16, CD32, and CD64 in tumor cell lines. We found that CD32 was highly expressed in K562 cells, whereas there was no detectable expression of CD16 or CD64 (Fig. 4A). We next determined whether cytolytic activity of 6B11 mAb-treated NKT cells was dependent on CD32. We cocultured iNKT cells treated with the 6B11 mAb and K562 cells in the presence or absence of an anti-CD32 mAb, which was previously reported to be a blocking antibody (15). When iNKT cells were cocultured with K562 cells in the presence of the anti-CD32 mAb, CD107a^+^ iNKT cells remarkably decreased from 52.8% to 1.8%, suggesting that degranulation of iNKT cells involving the 6B11 mAb was dependent on CD32 (Fig. 4B). We further examined CD32 expression in cell lines other than K562 cells to address whether CD32 expression correlated to with cytotoxic activity of iNKT cells treated with the 6B11 mAb. U937 and Daudi cells highly expressed CD32, but A549, PC13, and Jurkat cells did not express CD32 (Fig. 4C). Because iNKT cells treated with the 6B11 mAb were cytotoxic to U937 and Daudi cells, but not A549, PC13, or Jurkat cells (Fig. 3B), CD32 expression positively correlated to cytotoxicity of iNKT cells treated with the 6B11 mAb. We next determined whether CD32 overexpression in CD32-negative A549 cells induced cytotoxic activity of 6B11 mAb-bound iNKT cells. To this end, we generated A549 cells expressing CD32 at various levels (low, medium, and high) by lentiviral transduction (Fig. 4D). When iNKT cells treated with the 6B11 mAb were cocultured with CD32^+^ A549 cells, degranulation of iNKT cells was induced in accordance with the CD32 expression level in A549 cells (Fig. 4E). To determine whether FC receptors other than CD32 induced degranulation of 6B11 mAb-bound iNKT cells, we examined U937 cells as the targets of iNKT cells. Because U937 cells expressed CD32 and CD64, but not CD16 (Fig. 4C and F), we determined whether cytolytic activity of 6B11 mAb-bound NKT cells was dependent on CD32 or CD64. We cocultured iNKT cells treated with the 6B11 mAb and U937 cells in the presence of the anti-CD32 mAb, anti-CD64 mAb, or mIgG1 as a control. When the anti-CD32 mAb was added during coculture, CD107a^+^ iNKT cells were decreased from 38.3% to 15.6%, whereas blockade of CD64 by anti-CD64 mAb treatment did not affect degranulation of iNKT cells (Fig. 4G). These data indicated that iNKT cells treated with the 6B11 mAb were cytotoxic to U937 cells in a manner dependent on CD32, but not CD64.

### NK Cell–activating Receptors and Costimulatory Molecules are Involved in Degranulation of 6B11 mAb-bound iNKT Cells Toward K562 Cells

**FIGURE 5:**
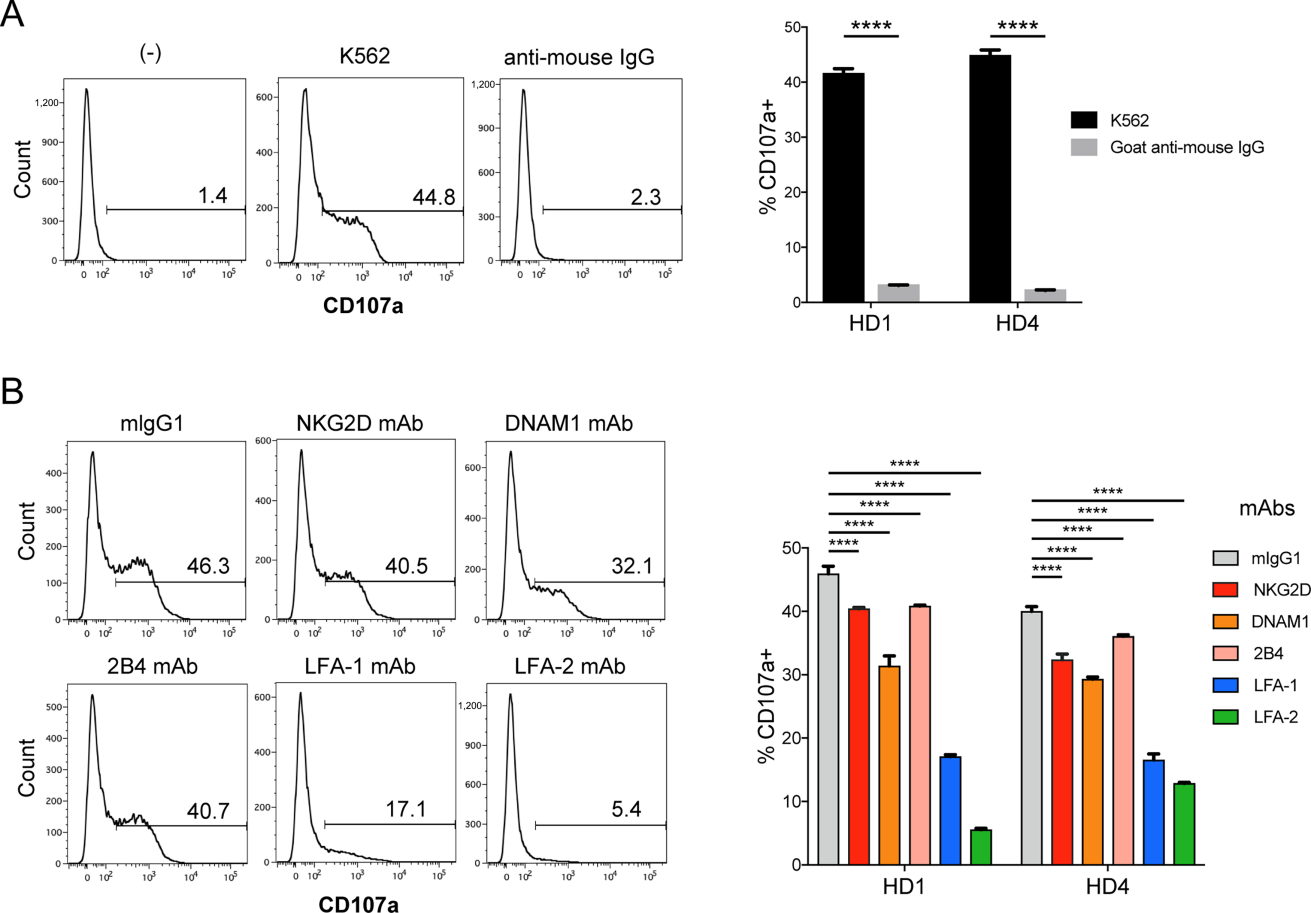
NK cell–activating receptors and costimulatory molecules are involved in degranulation of 6B11-mAb bound iNKT cells toward K562 cells. **A** and **B,** iNKT cells sorted using Vα24-FITC Ab/FITC MicroBeads were maintained in the presence of IL2 overnight, washed twice, and then cultured in fresh medium with IL2 for 4 or 5 days. **A,** iNKT cells treated with the 6B11 mAb (50 ng/mL) overnight were washed and cross-linked with goat anti-mouse IgG (10 µg/ mL) or cocultured with K562 cells (control). A CD107a assay was then performed. **B,** iNKT cells treated with the 6B11 mAb (50 ng/mL) overnight were washed and cocultured with K562 cells at an E/T ratio of 2:1 in the presence of anti-NKG2D, anti-DNAM1, anti-2B4, anti-LFA-1, anti-LFA-2, or isotype (mIgG, 10 µg/mL) Abs. A CD107a assay was then performed. Data are representative of two independent experiments. Data represent the mean ± SEM. ****, *P* < 0.0001. HD, healthy donor.

Our data indicated that the 6B11 mAb attached to CD32 induced cross-linking of TCR complexes on iNKT cells that were cytolytic toward CD32^+^ tumor cells. We next assessed whether cross-linking of TCRs expressed in iNKT cells bound to the 6B11 mAb was sufficient to induce degranulation of iNKT cells. 6B11 mAb-bound iNKT cells degranulated toward K562 cells, whereas cross-linking of 6B11 mAb-bound iNKT cells using goat anti-mouse IgG did not induce degranulation, indicating that iNKT cells required TCR cross-linking as well as other activation signals for degranulation (Fig. 5A). We next assessed whether NK cell–activating receptors, such as NKG2D, DNAM1, and 2B4, and costimulatory molecules LFA-1 and LFA-2 were involved in this process by treatment with blocking Abs for each molecule as reported previously (11, 12). Blockade of NKG2D, 2B4, and DNAM on iNKT cells led to a significant decrease in degranulation toward K562 cells by iNKT cells (Fig. 5B). Blockade of LFA-1 and LFA-2 more drastically decreased the degranulation toward K562 cells by iNKT cells compared with that of NK cell–activating receptors. These data suggested that cross-linking of 6B11 mAb-bound iNKT cells required activation signals other than the TCR signal for degranulation. However, we confirmed that certain methods to stimulate TCRs, such as plate-bound 6B11 and anti-CD3ε Abs, but not an α-GalCer-loaded CD1d tetramer, were capable of inducing degranulation of iNKT cells (Supplementary Fig. S2)

### CD32 Expressed by Primary Tumor Cells Induces Degranulation of 6B11 mAb-bound iNKT Cells

**FIGURE 6:**
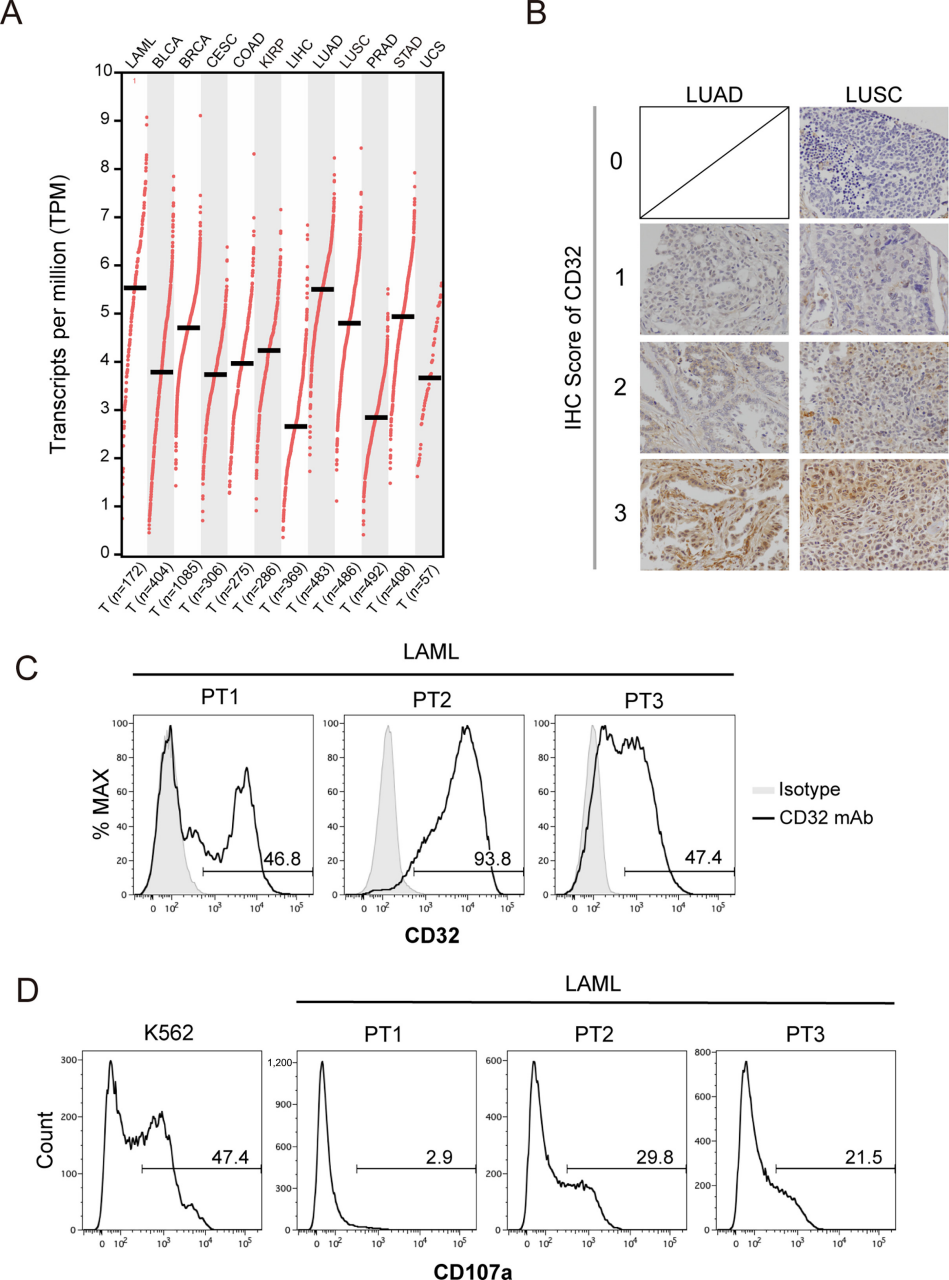
Primary tumor cells express CD32. **A,** Expression levels of *FCGR2A* across TCGA tumors were assessed by GEPIA. TCGA study abbreviations: LAML, acute myeloid leukemia; BLCA, bladder urothelial carcinoma; BRCA, breast invasive carcinoma; CESC, cervical squamous cell carcinoma and endocervical adenocarcinoma; COAD, colon adenocarcinoma; KIRP, kidney renal papillary carcinoma; LIHC, liver hepatocellular carcinoma; LUAD, lung adenocarcinoma; LUSC, lung squamous cell carcinoma; PRAD, prostate adenocarcinoma; STAD, stomach adenocarcinoma; UCS, uterine carcinosarcoma. **B,** Lung tumor tissue arrays were subjected to IHC to detect CD32 expression in LUAD and LUSC (×400). **C,** LAML cells were stained with anti-CD32 or isotype control Abs and analyzed by flow cytometry. **D,** iNKT cells sorted using Vα24-FITC Ab/FITC MicroBeads were maintained in the presence of IL2 overnight, washed twice, and then cultured in fresh medium with IL2 for 4 or 5 days. iNKT cells treated with the 6B11 mAb (50 ng/mL) overnight were washed and cocultured with LAML or K562 cells (control) at an E/T ratio of 2:1. A CD107a assay was then performed. PT, patient.

We assessed whether primary tumor cells expressed CD32 by GEPIA. CD32 has three isoforms, namely A–C. We analyzed expression of the *FCGR2A* gene, which encodes the major isoform, CD32A. We chose 12 cancers from the database, all of which expressed *FCGR2A* (Fig. 6A). We confirmed CD32 expression in lung adenocarcinoma and lung squamous cell carcinoma by IHC (Fig. 6B; Table 1). We also confirmed CD32 expression in LAML by flow cytometry (Fig. 6C). When iNKT cells treated with 6B11 mAb were cocultured with LAML cells, we observed degranulation of iNKT cells toward LAML cells in 2 out of 3 donors, although the proportion of CD107a^+^ iNKT cells was lower in coculture with LAML cells compared with that in K562 cells (Fig. 6D). These data suggested that 6B11 mAb-bound iNKT cells exerted cytolytic activity toward CD32^+^ primary tumor cells.

**TABLE 1:** IHC scoring of CD32 in the patients with lung cancer

P-Score	LUAD *n* (%)	LUSC *n* (%)
0: negative	0 (0)	1 (4.3)
1: positive <10%	7 (26.9)	10 (43.5)
2: positive 10∼50%	9 (34.6)	7 (30.4)
3: positive >50%	10 (38.5)	5 (21.7)
Total	26 (100)	23 (100)

Abbreviations: LUAD, lung adenocarcinoma; LUSC, lung squamous cell carcinoma; P-Score; proportional score.

### CD32-expressing Normal Cells Are Not Targets of 6B11 mAb-bound iNKT Cells

**FIGURE 7:**
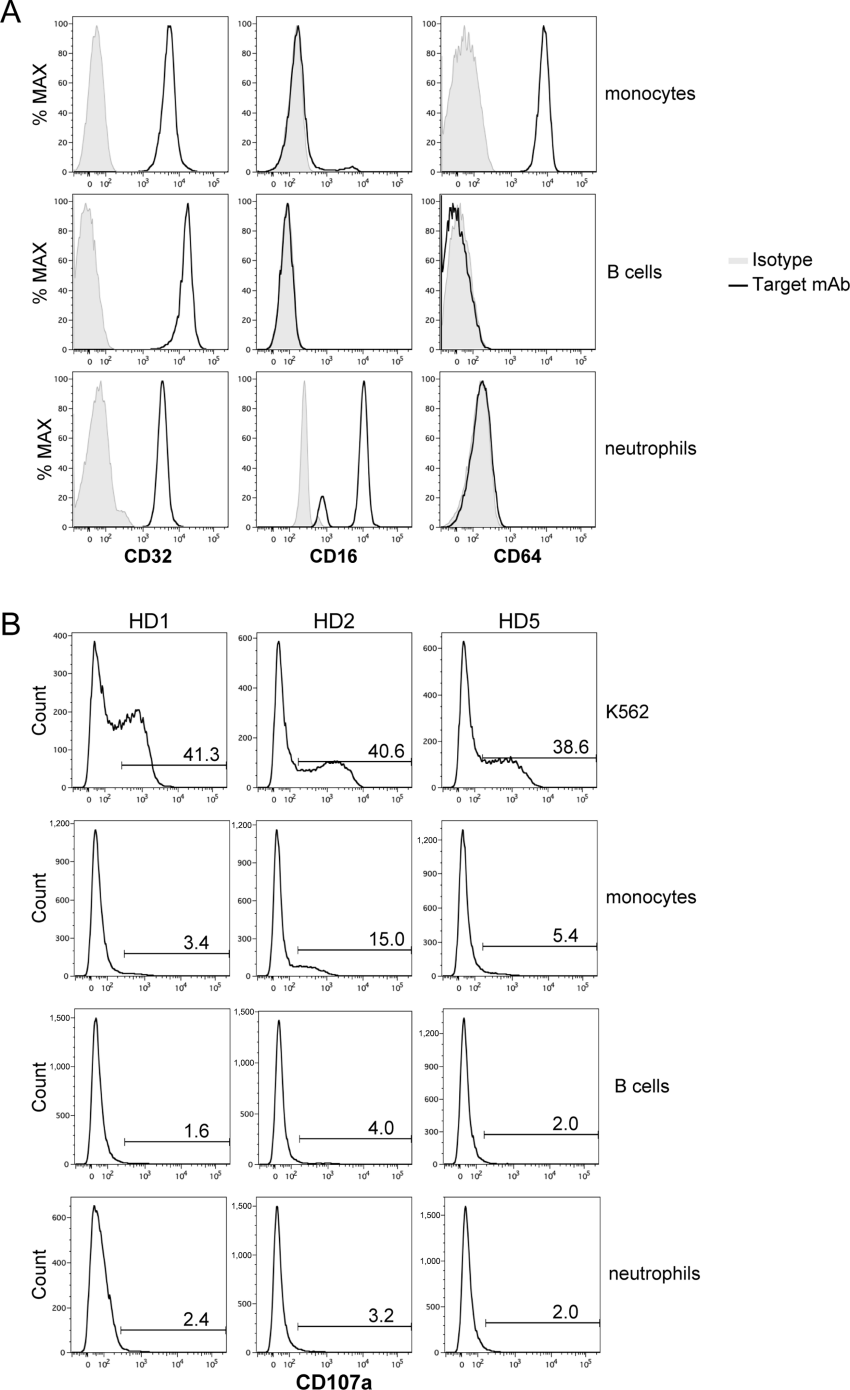
CD32-expressing normal cells barely induce degranulation of 6B11 mAb- bound NKT cells. **A,** PBMCs isolated from healthy donors were stained with Abs against FC receptors (CD16, CD32, and CD64), a monocyte marker (CD14), B-cell marker (CD19), and neutrophil marker (CD66b). Stained cells were analyzed by flow cytometry. Data are representative of three independent experiments. **B,** iNKT cells sorted using Vα24-FITC Ab/FITC MicroBeads were maintained in the presence of IL2 overnight, washed twice, and then cultured in fresh medium with IL2 for 4 or 5 days. iNKT cells treated with the 6B11 mAb (50 ng/mL) overnight were washed and cocultured with monocytes, B cells, neutrophils, or K562 cells (control) at an E/T ratio of 2:1. A CD107a assay was then performed. HD, healthy donor.

We demonstrated that primary tumor cells expressed CD32. However, normal immune cells also express CD32. Therefore, we determined whether 6B11-mAb bound iNKT cells degranulated toward normal immune cells, including monocytes, B cells, and neutrophils. We assessed the expression of FC receptors CD32, CD16, and CD64 by flow cytometry (Fig. 7A). All monocytes, B cells, and neutrophils highly expressed CD32. Moreover, monocytes coexpressed CD64 and neutrophils coexpressed CD16. When 6B11 mAb-bound iNKT cells were cocultured with monocytes, B cells, and neutrophils, CD107a^+^ iNKT cells were barely detectable except when cocultured with monocytes of healthy donor 2, indicating minimal off-target effects of 6B11 mAb treatment on iNKT cells to exhibit cytolytic activity (Fig. 7B).

### 6B11 mAb Treatment Enhances Antitumor Immunity of iNKT Cells Toward K562 Cells *In Vivo*

**FIGURE 8:**
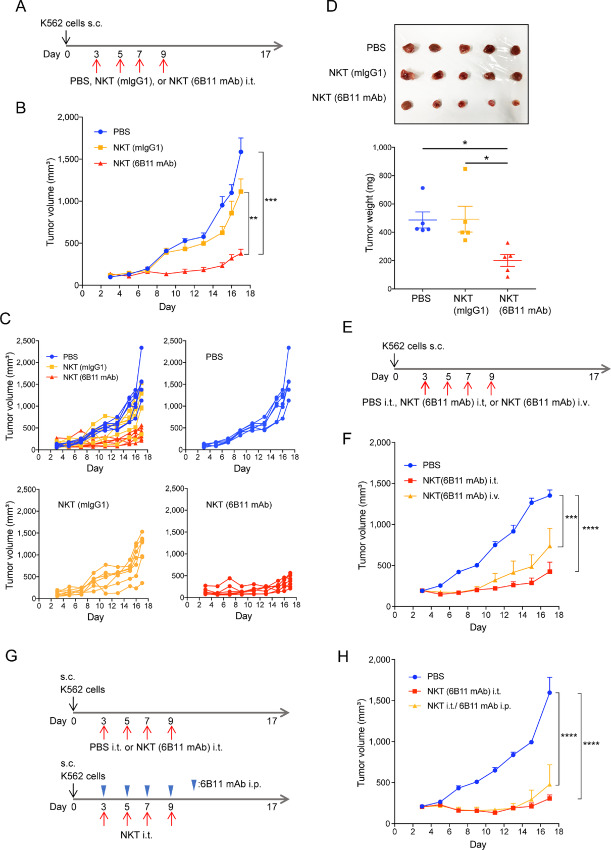
6B11 mAb-treated iNKT cells suppress tumor growth *in vivo*. **A,E,G,** Schematic of iNKT cell therapy *in vivo*. **B**–**D,** Human IL7/IL15 knockin NSG mice were subcutaneously injected with K562 cells on day 0. iNKT cells treated with the 6B11 mAb (50 ng/mL) or mIgG1(50 ng/mL) overnight were washed and resuspended in PBS. **B,** iNKT cells were intratumorally injected on days 3, 5, 7, and 9 (*n* = 6 or 7 mice/group). **B** and **C,** Tumor size was measured every 2 days. **D,** Tumors were excised at day 13 and their weight was measured. **F,** Human IL7/IL15 knock-in NSG mice were subcutaneously injected with K562 cells on day 0. iNKT cells treated with the 6B11 mAb as described in B were intratumorally or intravenously injected on days 3, 5, 7, and 9 (*n* = 5 or 6 mice/group). **H,** Human IL7/IL15 knock-in NSG mice were subcutaneously injected with K562 cells on day 0. iNKT cells treated with the 6B11 mAb as described in B or untreated iNKT cells were intratumorally injected on days 3, 5, 7, and 9 (*n* = 5 or 6 mice/group). The 6B11 mAb was further intraperitoneally administrated to mice injected with untreated NKT cells. Data are representative of two independent experiments. Data represent the mean ± SEM. *, *P* < 0.05; **, *P* < 0.01; ***, *P* < 0.001; ****, *P* < 0.0001.

To determine whether 6B11 mAb treatment of iNKT cells could be a therapeutic approach, we next investigated the effect of the 6B11 mAb in a K562 xenograft mouse model *in vivo*. hIL7 × hIL15 KI NSG mice were used to constantly supply human IL7 and IL15 for iNKT cell survival in mice. NSG mice subcutaneously injected with K562 cells were intratumorally injected with iNKT cells treated with the 6B11 mAb or control mIgG1 at days 3, 5, 7, and 9, and tumor size was monitored (Fig. 8A). The number of doses was determined in previous studies (13, 16). Because NSG mice lack functional immune cells, we could assess the direct effect of iNKT cells on K562 cells *in vivo*. Mice that received iNKT cells treated with the 6B11 mAb exhibited repressed tumor growth compared with mice that received mIgG1-treated iNKT cells (Fig. 8B and C). There was no effect of mIgG1-treated iNKT cells compared with the PBS injection control. The mean tumor weight was significantly lower in mice that received iNKT cells treated with 6B11 mAb compared with control mice (Fig. 8D). When 6B11 mAb-bound iNKT cells were injected intravenously instead of intratumorally, tumor growth was suppressed in the same manner as intratumoral injection of 6B11 mAb-bound iNKT cells (Fig. 8E and F). We further examined whether iNKT cells exhibited antitumor immunity when iNKT cells were intratumorally injected and the 6B11 mAb was intraperitoneally injected separately. Mice that received iNKT cells and the 6B11 mAb separately also showed suppression of tumor growth (Fig. 8G and H). These data suggested that 6B11 mAb treatment enhanced antitumor immunity of iNKT cells toward K562 cells *in vivo*.

## Discussion

Here, we report the mechanism by which iNKT cells exert cytotoxicity toward CD1d-negative K562 cells in a TCR-dependent manner. In our previous study, we found that TCRs expressed on iNKT cells were required to target CD1d-negative K562 cells. In contrast to this observation, others reported that iNKT cells cannot target CD1d-negative tumor cells, because iNKT cells express an invariant TCR that recognizes antigens presented on CD1d (5). In this study, we demonstrated that an anti-Vα24 mAb carried over from positive sorting mediated the cytotoxic activity of iNKT cells toward CD1d-negative K562 cells. Moreover, we found that the FC portion of the anti-Vα24 mAb attached to iNKT cells was bound to CD32 on K562 cells, thereby inducing cross-linking of TCRs on iNKT cells. Treatment with another anti-iNKT TCR mAb, 6B11 mAb, also induced cytotoxic activity of iNKT cells toward CD1d-negative K562 cells *in vitro* and *in vivo*. Therefore, cytotoxic activity toward CD1d-negative K562 was thought to be fully dependent on carried over anti-iNKT-TCR antibodies in previous studies. We speculated that the amount of carried over anti-iNKT-TCR antibody among different experimental settings created the discrepancy in the cytotoxic activity of iNKT cells toward CD1d-negative tumor cells. While the number of iNKT cells is very low among human PBMCs, which need to be expanded prior to experiments to assess the functions of iNKT cells such as cytotoxic activity, we need to aware that Abs used for either sorting or expansion may skew the cytotoxic activity of iNKT cells toward CD1d-negative tumor cells.

Escriba-Garcia and colleagues recently reported that a mouse iNKT cell Ab, NKT14, induces an antitumor response against B-cell lymphoma *in vivo* (17). These data may fit our model if FC receptors are expressed in B-cell lymphoma cell line 4TOO for CD32-dependent cytotoxic activity of iNKT cells.

Antibody-dependent cell-mediated cytotoxicity (ADCC) is an antitumor mechanism that is mainly mediated by NK cells (18, 19). NK cells recognize and kill antibody-bound tumor cells through FC receptor III (CD16) on NK cells. Our data represent a case of reverse ADCC, because target tumor cells express FCγ receptor II (CD32) and the FC portion of mAb is bound to CD32 at the tumor site, while the mAb recognizes iNKT TCRs. Therefore, iNKT cells require an anti-iNKT TCR mAb to recognize and kill CD1d-negative tumor cells via CD32 in a reverse ADCC-dependent manner. However, activation of the TCR complex by the 6B11 mAb-CD32 axis was not sufficient to induce cytotoxic activity, and NK cell–activating receptors and costimulatory molecules are involved in this event, because cross-linking of iNKT TCRs using the anti-iNKT TCR mAb with anti-mouse Ig did not induce cytotoxic activity of iNKT cells. Our data also demonstrated that iNKT cells treated with the 6B11 mAb were cytotoxic toward primary tumor cells of LAML. In contrast to primary tumor cells, normal immune cells, which express CD32, barely induced degranulation of iNKT cells, indicating that anti-iNKT TCR mAb-bound iNKT cells recognize and target tumors specifically without affecting normal cells.

mAbs are widely used in cancer immunotherapy (20, 21). mAbs, which recognize tumor-specific antigens are enable to elicit target therapy whereas systemic chemotherapy or radiotherapy often cause broad side effects (22). Some mAbs that induce ADCC are approved by the FDA, such as anti-GD2, anti-CD20, and anti-CCR4 Abs (23–26). Our data suggest the application of anti-iNKT TCR mAbs in the clinic to treat CD32^+^ tumors, such as leukemia and lymphoma, by inducing reverse ADCC, although humanization of anti-iNKT TCR mAbs might be needed. Whereas anti-GD2, anti-CD20 mAbs are known to induce the off-target effects due to expression of their antigen on normal cells, anti-iNKT TCR mAbs have a potential to overcome these issues. As described above, activation signaling induced by anti-iNKT TCR mAbs is not sufficient enough to elicit cytotoxic activity of iNKT cells, thus minimizing off target effect. The 6B11 mAb has been assessed in a phase I clinical trial of immunotherapy for advanced melanoma to sort iNKT cells prior to expansion (7). Our model supports that the 6B11 mAb can be applied to treat CD32^+^ tumors such as leukemia, lymphoma, and lung cancer (27, 28).

In this study, we focused on CD32 expression because K562 cells expressed CD32, but did not express other FC receptors such as CD16 and CD64. Further study will be needed to address whether CD16 and CD64 are involved in cytotoxic activity of iNKT cells treated with the 6B11 mAb in the same manner as CD32 to target other tumor cells. In our *in vivo* experimental setting, we addressed the direct interaction between iNKT cells and CD1d-negative tumor cells using immunodeficient mice which does not contain other immune cells such as NK cells. Therefore, we could not determine whether iNKT cells treated with an anti-iNKT TCR mAb could be the target of ADCC from NK cells. The balance between reverse ADCC targeting tumor cells by iNKT cells and ADCC targeting iNKT cells by NK cells and other effector cells also needs to be examined in a future study.

Overall, our data demonstrate how anti-iNKT TCR mAb-bound iNKT cells recognize and kill CD1d-negative target tumors. The anti-iNKT TCR mAb is bound to CD32 at the tumor site, thereby bridging iNKT cells and CD1d-negative tumors. These finding shed light on the therapeutic potential of anti-iNKT TCR mAbs in NKT cell–based immunotherapy to treat CD1d-negative CD32^+^ cancers.

## Supplementary Material

Supplementary Figure 1 Cytotoxic activity of iNKT cells toward K562 cells gradually decreases daily.

Supplementary Figure 2 Plate-bound 6B11 or anti-CD3 mAb stimulation induces degranulation of iNKT cells.
