# Supplementary material for: Anti-Vα24Jα18 TCR Antibody Tunes iNKT Cell Responses to Target and Kill CD1d-negative Tumors in an FcγRII (CD32)-dependent Manner
Source: Cancer Res Commun. 2024 Feb 19;4(2):446–59. doi: 10.1158/2767-9764.CRC-23-0203 (PMC10875981; doi:10.1158/2767-9764.CRC-23-0203)
Supplement: Supplementary Figure 1 — Cytotoxic activity of iNKT cells toward K562 cells gradually decreases daily. [file crc-23-0203-s01.pdf]

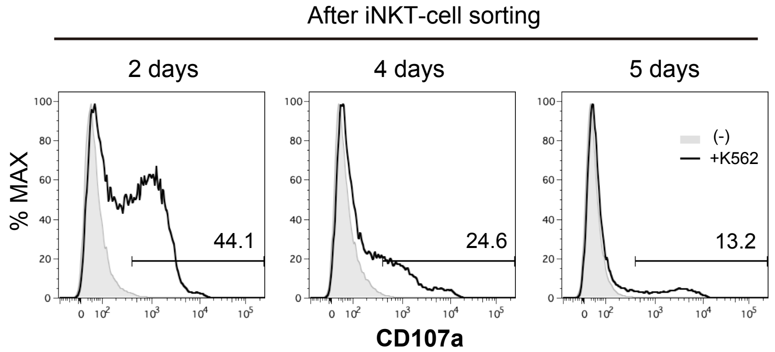

Supplementary Fig. S1 Cytotoxic activity of iNKT cells toward K562 cells gradually decreases daily. PBMCs isolated from healthy donors were cultured in the presence of α-GalCer and IL-2 for 9–10 days, followed by sorting using Vα24-FITC Ab/FITC MicroBeads. iNKT cells were then treated with IL-2 until analysis. iNKT cells maintained for 2 days, 4 days, or 5 days after sorting were cocultured with K562 at an E/T ratio of 2:1 and then a CD107a assay was performed.
