# Supplementary material for: Anti-Vα24Jα18 TCR Antibody Tunes iNKT Cell Responses to Target and Kill CD1d-negative Tumors in an FcγRII (CD32)-dependent Manner
Source: Cancer Res Commun. 2024 Feb 19;4(2):446–59. doi: 10.1158/2767-9764.CRC-23-0203 (PMC10875981; doi:10.1158/2767-9764.CRC-23-0203)
Supplement: Supplementary Figure 2 — Plate-bound 6B11 or anti-CD3 mAb stimulation induces degranulation of iNKT cells. [file crc-23-0203-s02.pdf]

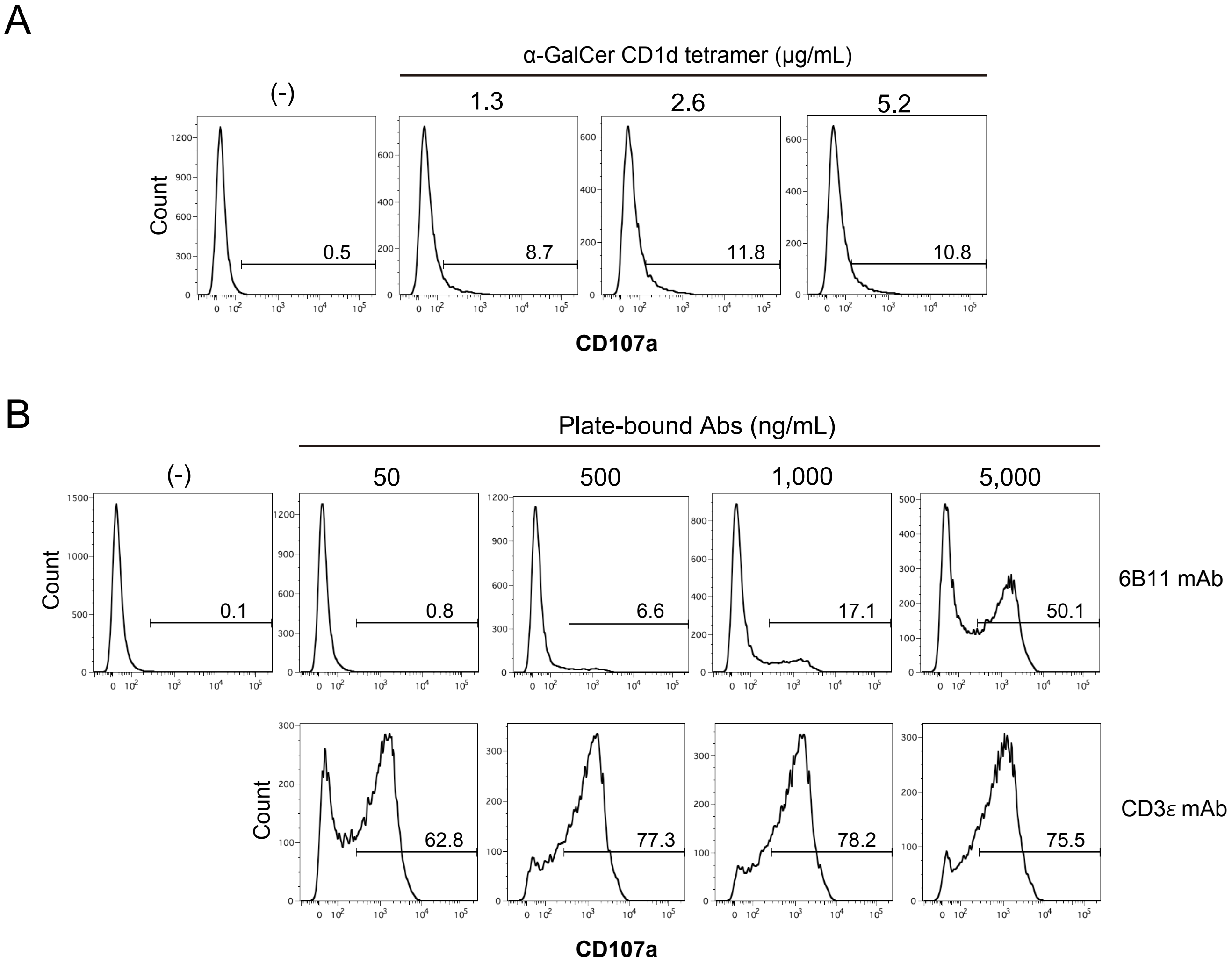

Supplementary Fig. S2 Plate-bound 6B11 or anti-CD3 mAb stimulation induces degranulation of iNKT cells. (A, B) iNKT cells sorted using Vα24-FITC Ab/FITC MicroBeads were maintained in the presence of IL-2 overnight, washed twice, and then cultured in fresh medium with IL-2 for 4 or 5 days. (A) iNKT cells were stimulated with an α-GalCer-loaded CD1d tetramer at the indicated concentrations for 2 hours. A CD107a assay was then performed. (B) iNKT cells were stimulated with plate-bound 6B11 or CD3 mAbs for 2 hours. A CD107a assay was then performed. Data are representative of two independent experiments.
